# Deficient Decision Making in Pathological Gamblers Correlates With Gray Matter Volume in Medial Orbitofrontal Cortex

**DOI:** 10.3389/fpsyt.2020.00109

**Published:** 2020-03-04

**Authors:** Daniel Freinhofer, Philipp Schwartenbeck, Natasha Thon, Tina Eigenberger, Wolfgang Aichhorn, Melanie Lenger, Friedrich M. Wurst, Martin Kronbichler

**Affiliations:** ^1^ Centre for Cognitive Neuroscience, University of Salzburg, Salzburg, Austria; ^2^ Neuroscience Institute, Christian-Doppler Medical Centre, Paracelsus Medical University Salzburg, Salzburg, Austria; ^3^ Wellcome Trust Centre for Human Neuroimaging, University College London, London, United Kingdom; ^4^ Oxford Centre for Functional MRI of the Brain, Nuffield Department of Clinical Neurosciences, University of Oxford, Oxford, United Kingdom; ^5^ Department of Psychiatry, Psychotherapy and Psychosomatics, Christian-Doppler Medical Centre, Paracelsus Medical University, Salzburg, Austria; ^6^ Department for Psychiatry and Psychotherapy, Medical University of Graz, Graz, Austria; ^7^ Medical Faculty and Psychiatric University Hospital, University of Basel, Basel, Switzerland

**Keywords:** pathological gambling, decision making, cups task, impulsivity, magnetic resonance imaging, voxel-based morphometry

## Abstract

Individuals suffering from pathological gambling (PG) show impaired decision making, but it is still not clear how this impairment is related to other traits and neuroanatomical characteristics. In this study, we investigated how the influence of PG on decision making (1) is connected to different impulsivity facets and (2) how it is related to gray matter volume (GMV) in various brain regions. Twenty-eight diagnosed PG patients and 23 healthy controls completed the cups task to measure decision making. In this task, participants had to decide between safe and risky options, which varied in expected value (EV) between risk advantageous, equal EV, and risk disadvantageous choices. A delay discounting task and the Barrant Impulsiveness Scale were applied to assess multiple impulsivity facets. In addition, structural magnetic resonance images were acquired. In comparison to the control group PG patients demonstrated more deficits in decision making, indicated by less EV sensitivity, but there was no significant difference in number of overall risky choices. Also, PG patients showed increased impulsivity in nearly every dimension. Results revealed (1) a positive correlation between decision making impairments and non-planning impulsivity but no significant relation to other impulsivity facets. Although we found no GMV differences between PG patients and controls, (2) a regions of interest analysis showed a correlation between medial orbitofrontal GMV and EV sensitivity in PG patients. Our findings showed that (1) the association between decision making and impulsivity can also be found in PG patients, but only for certain impulsivity facets. This suggests that it is essential to consider measuring different dimensions, when investigating impulsivity in a PG sample. Secondly, our findings revealed that (2) dysfunctional decision making—particularly the component of risk evaluation—is related to decreased GMV in the medial orbitofrontal cortex, a brain region concerned with processing of rewards. Interestingly, we did not find more risky choices for PG patients, and thus, we assume that decision making deficits in PG are primarily related to risk evaluation, not risk seeking, which is in line with our GMV findings.

## Introduction

In the recently accepted eleventh version of the International Classification of Diseases (ICD-11) as well as in the Diagnostic and Statistical Manual of Mental Disorders 5 (DSM 5), gambling disorder or pathological gambling (PG) is classified as an addictive disorder (1, 2). In the ICD-11, gambling disorder is defined as a disorder, that consists of persistent or recurrent patterns of gambling behavior, which are characterized by impaired control over gambling, increased priority given to gambling over other life interests, and the continuation of gambling despite negative consequences, that result in significant impairment in personal, family, occuppational or other areas of functioning (2). In the DSM five criteria for PG include attempts to “chase” one's losses (i.e., return to gambling after losing to get even), and unsuccessful efforts to control, cut back, or stop gambling (1). Thus, the phenomenology of PG according to both classifications is in part characterized by symptoms that reflect impaired decision making. This relationship between PG and decision making is also supported by several studies, which demonstrated that patients suffering from PG show poorer decision making abilities (3–8). There is sufficient evidence on this relationship, but there are still less researched issues in relation to decision making in PG.

### Decision Making and Impulsivity in PG

Besides decision making deficits, pathological gamblers also display an increased level of impulsivity (4, 7, 9–12) and in studies investigating non-clinical samples higher impulsivity measures usually correlates with impaired decision making (13–16). However, we found only two studies investigating the relation between decision making and impulsivity in a PG sample and their methodical approaches and results differed considerably (8, 17). In the study by Nigro and Cosenza (8), adolescent gamblers and non-gamblers completed the Iowa Gambling Task [IGT, (18)], which is used as measure for decision making, and a delay discounting task, which is regarded as a measure for behavioral impulsivity. They found a negative correlation between the IGT score and the delay discounting parameter, but only for the non-gambling group. Whereas Kräplin et al. (17) obtained several decision making parameters via the Cambridge Gambling Task (19) as well as behavioral and self-reported impulsivity measures and discovered a positive correlation between delay discounting and impaired decision making components, which was solely driven by the pathological gamblers. They also reported a positive correlation between impaired decision making components and a subscale of self-reported impulsivity for both the PG and control group.

Studies with other clinical but non-gambling samples also showed ambiguous results in regard to the relation between decision making and impulsivity. Since PG was recently categorized as a “Substance-Related and Addictive Disorder” in the DSM 5, we primarily considered studies with patients suffering from substance-related disorders. In cocaine-dependent samples, there was no correlation between IGT score and self-reported impulsivity scores (20, 21), but significant correlation between IGT performance and behavioral impulsivity measured by delay discounting (20). In a mixed sample of alcohol and stimulant dependent patients on the other hand, a connection was found between IGT score and self-reported impulsivity (22). Furthermore, a study on eating disorder also investigated the association between decision making and impulsivity and reported a correlation between self-reported impulsivity and IGT score for the subgroup of participants suffering from the binge/purging type of eating disorder (23).

To summarize, these studies exploring PG or similar disorders give no clear indication on the association between decision making and impulsivity in pathological gamblers. This might be the result of different dimensions of impulsivity and various components of decision making that were assessed in these studies. Hence, to increase the understanding of the link between decision making and impulsivity in pathological gamblers, we intend to measure one concrete component of decision making and investigate its correlation with a variety of impulsivity dimensions. Behavioral impulsivity was measured by a delay discounting task and self-reported impulsivity facets included motor, attentional, and non-planning impulsivity from the Barratt Impulsiveness Scale. Motor impulsivity is defined as taking actions without thinking; attentional impulsvivity represents lack of focus on the task at hand; and non-planning impulsivity is characterized as an orientation towards the present, rather than to the future (24). All these facets of impulsivity are not necessarily strongly correlated with each other (25) and might be differently related to impaired decision making (17).

Although a vast amount of gambling-related studies apply the IGT to measure impaired decision making and it is regarded as a good representation of real life decision situations (26), we decided against this task, because the IGT involves multiple components of the decision making process including preference formation and feedback processing (27). Thus, the IGT score is influenced by several processes, which can be differently affected by PG and differently related to impulsivity. A task which assesses a specific component of decision making is the cups task (28). The cups task applies decision making under risk and a person's sensitivity for different expected values (EVs) can be used as a measure for risk evaluation, a component of preference formation in decision making (27).

According to the cognitive framework on decision making by Bechara et al. (29), deficits in reward- and valuation-related components of decision making are positively correlated to cognitive impulsivity but not motor impulsivity. Cognitive impulsivity is characterized by behavior like delay discounting (30) or the non-planning impulsivity facet, whereas motor impulsivity refers to the inability to inhibit inappropriate responses (11). The role of attentional impulsivity is less clear and it is also not directly discussed in Bechara et al. (29), but there are studies showing, that attentional impulsivity is also less connected to decision making (31). Thus, in line with previous research (17), we assume, cognitive impulsivity is positively correlated with a valuation-related component of decision making, whereas motor impulsivity measures are not related to decision deficits in PG.

### Decision Making and Gray Matter Volume in PG

Another relatively less researched issue regarding decision making in PG are relations to neuroanatomical characteristics like gray matter volume (GMV). On the one hand, decision making deficits in addictive disorders like PG and substance use disorders are related to brain regions including the dorsolateral prefrontal cortex, the ventromedial prefrontal/orbitofrontal cortex, and the insula (32–36). On the other hand, PG patients showed reduced GMV in amygdala (37), putamen (38) hippocampal areas (38, 39), and various prefrontal regions (40, 41). However, we found no study investigating the association of decision making deficits and GMV in PG patients. One study though found a negative correlation between IGT scores and GMV in the medial orbitofrontal cortex in patients suffering from substance use disorder (35). In addition, decision making–related processes and GMV were examined in a PG sample by exploring gambling-related cognitive biases Ruiz de Lara et al. (41). A negative correlation was found between GMV in the dorsal anterior cingulate cortex and interpretative bias, which is defined as the tendency to reinterpret gambling outcomes (42).

In line with the results on decision making and GMV in participants with a PG-related disorder (35) and since we investigated the cups task and EV sensitivity, we decided to focus on the orbitofrontal cortex in relation to decision making in PG. Theoretical background for this decision was provided by a model of neural mechanisms underlying decision making in the prefrontal cortex by Wallis (43). According to this model, the orbitofrontal cortex integrates information from various sources (e.g., amygdala, hypothalamus, etc.) and calculates the EV of a potential outcome, which subsequently is used by medial and dorsolateral prefrontal cortices to determine if and which behavioral responses should be applied to obtain the outcome. Following this model, reduced orbitofrontal gray matter should be associated with impaired calculating of an EV for a potential outcome and therefore be associated with poorer performance in a task with decision under risk like the cups task.

Indeed, lesions in ventromedial/orbitofrontal areas are connected to less sensitivity for different EV levels in the cups task (44). Compared to lesions in insula or amygdala, which show decreased EV sensitivity more specific for either the loss and/or gain domain, the effect of lesions in the orbitfrontal cortex is more severe and domain-insensitive (45). Brain activation in orbitofrontal areas also correlates with performance in decision making (46).

Connecting to the previously discussed topic of impulsivity, Ruiz de Lara et al. (41) also investigated impulsivity measures and found a negative correlation between GMV in the right ventrolateral prefrontal cortex and a self-reported impulsivity facet. Similarly, a study only exploring amygdala and striatum volumes, reported a negative correlation between left amygdala GMV and impulsivity in a PG sample (37). Additionally, findings from a healthy sample showed that cognitive impulsivity is negatively correlated to GMV in the orbitofrontal cortex (47). Considering this finding in relation to the association between cognitive impulsivity and decision making Bechara et al. (29), this finding is in line with our assumption that deficient decision making is connected with GMV in the orbitofrontal cortex (48).

Altough the dorsolateral prefrontal cortex also plays an important role in decision making processes (3), we did not include it in our specific regions of interest (ROIs), because, compared to medial orbitofrontal regions, it is not particularly connected to the calculation of subjective value of choice alternatives (49). In their meta analysis, Bartra et al. (49) distinguished between one set of regions including the dorsolateral prefrontal cortex and the insula connected to arousal or salience of subjective value, and another set of regions forming a valuation system consisting of ventromedial prefrontal cortex and ventral striatum. Further, they reported that the ventromedial prefrontal cortex is more related to value processing during the decision than in the outcome stage. Since we conducted the cups task without displaying outcomes, our measure of EV sensitivity is not influenced by effects of monetary feedback. Thus, we also exluded the ventral striatum from our ROIs and focused especially on the ventromedial prefrontal ROI from Bartra et al. (49) consisting of the gyrus rectus and medial orbitofrontal gyrus.

### Aims and Hypotheses

In summary, the aims of the present study were to extend the understanding of decision making—concretely, the decision making component of risk evaluation—in individuals suffering from PG and its relation to different facets of impulsivity as well as to GMV. In accordance to previous research, we expect deficits in decision making in PG patients compared to controls indicated by reduced sensitivity for EV differences, and we also expect the PG group to show higher levels of behavioral and self-reported impulsivity as well as sensation seeking. As our first main hypothesis, we assume, that measures of cognitive impulsivity, namely, delay discounting and the self-reported facet of non-planning impulsivity correlate negatively with our measure of decision making in the PG group. Regarding decision making and neuroanatomy in PG, we expect, that PG patients show reduced GMV in the orbitofrontal cortex. And finally, our second main hypothesis suggests, that increased impairment of decision making is correlated with decreased GMV in the orbitofrontal cortex. To our knowledge, this study, for the first time, investigates the association between GMV and decision making abilities in a PG sample.

## Materials and Methods

### Participants

This study has been approved by the local ethics committee (ethics committee of the Federal State Salzburg, Number 415-E/1632/9-2013). Twenty-eight patients suffering from PG according to criteria of the Diagnostic and Statistical Manual of Mental Disorders IV [DSM-IV; (50)] and 23 healthy controls (HC) without history of neurological or psychiatric disorders, or history of gambling behavior participated in the study. PG patients were recruited from the Christian-Doppler Medical Centre in Salzburg, where they were in treatment for PG. Controls were recruited by word-of-mouth advertising and mailings, and were matched regarding age and sex with the PG patients.

### Measures

#### Cups Task

**Figure 1:**
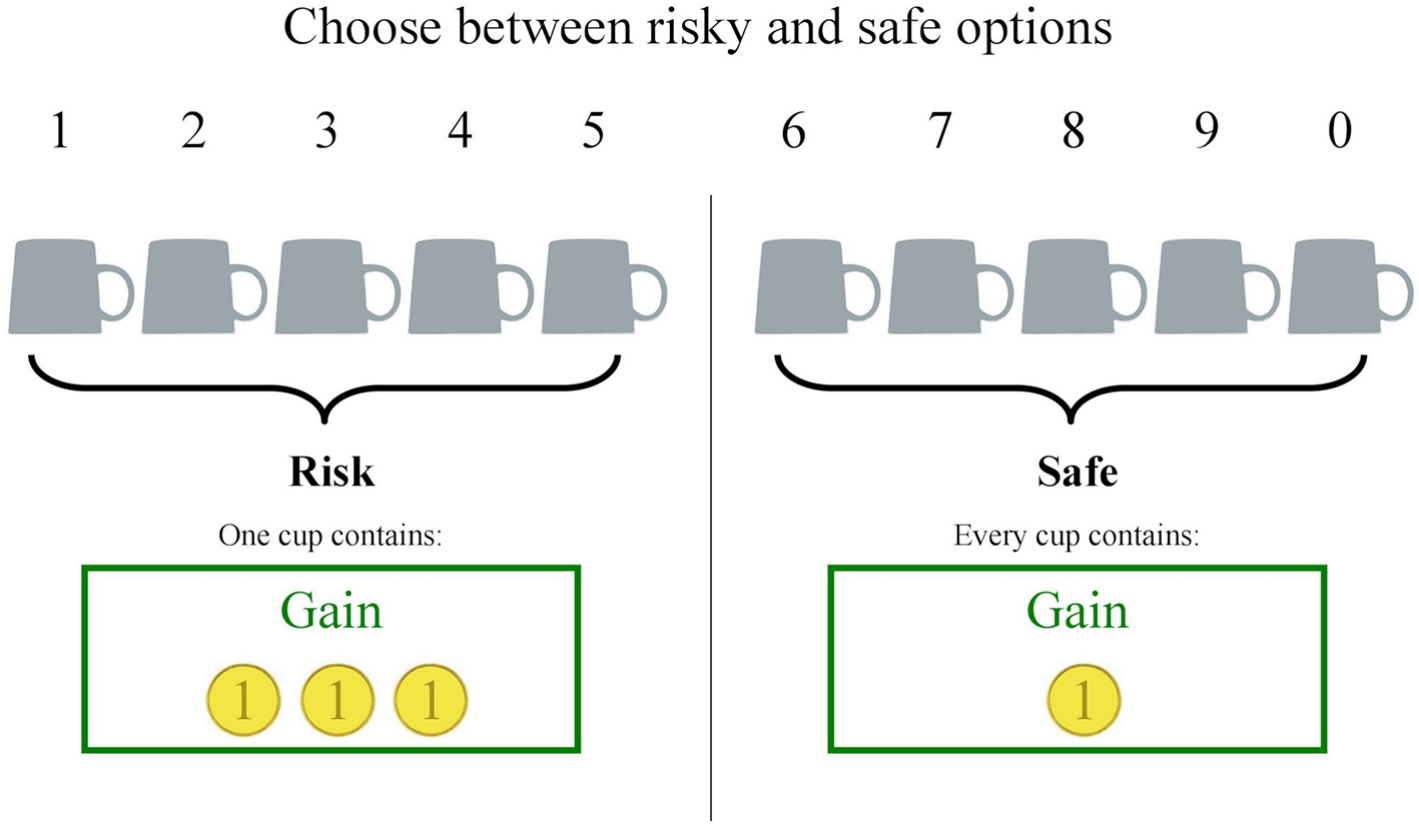
Task design of the cups task.

We used an adapted, computerized version of the cups task (51) to measure the quality in decision making. This task consisted of 54 trials in which participants had to choose between risky and riskless options presented on the left and right side of the screen. The options were represented by a varying amount of cups and coins. The amount of cups was always equal for both options per trial and varied between two, three, or five cups. In the riskless option, each cup always contained one coin (the depicted gold coins were not further described and had an undefined value. In the risky option, one of the cups contained the underneath depicted amount of coins (two, three, or five) while the others were empty. Thus, in the riskless option, there was a probability of 1 to choose a cup with one coin, while in the risky option the probability to choose the cup with the coins was 1/(amount of cups). Participants had to choose one of the cups either from the risky or riskless side by pressing the number button on the keyboard corresponding to the number above the chosen cup (see **Figure 1**). There was no time limit for the decision.

Trials were presented in two blocks, one where the coins represented monetary gains and one where the coins represented monetary losses. The order of block presentation was randomized. To describe good or bad decisions, the EV was used. EV is defined as the product of magnitude of the monetary outcome and the probability of receiving this outcome. The EV of the riskless option always equaled 1 in gain trials and −1 in loss trials, but the EV for the risky option was either higher, equal, or lower than the EV of the riskless option. This resulted in trials were the choice of the risky option was either advantageous, equal, or disadvantageous in comparison to choosing the riskless option (see **Table 1**).

**Table 1:** Different expected value (EV) categories for the risky option in the cups task according to probability (*P*) and magnitude (Coins).

	Gain domain		Loss domain	
	*P*	Coins	*P*	Coins
Risk advantageous EV	.33	5	.20	2
	.50	3	.20	3
	.50	5	.33	2
Risk equal EV	.20	5	.20	5
	.33	3	.33	3
	.50	2	.50	2
Risk disadvantageous EV	.20	2	.33	5
	.20	3	.50	3
	.33	2	.50	5

#### Delay Discounting Task

The delay discounting task (DDT) applied in this study was adapted from Pine et al. (52) and was used to obtain a behavioral measure of impulsivity. Participants had to choose between a smaller-sooner and a larger-later option in 200 trials. Each option consisted of a monetary outcome (varying between 1€ and 100€) and a corresponding delay time (varying between 1 week and 52 weeks) after which the outcome would be received. The options were presented left and right of a fixation cross and participants had to select via right or left mouse button which option they would prefer. No money was paid for real and participants were instructed that the monetary outcomes were only hypothetical. This method was used, because several studies showed that delay discounting paradigms with real or hypothetical rewards produced similar effects (53, 54). Additionally, 20 catch trials with a larger-sooner and a smaller-later option were included to check if the participants were paying attention to the choices. There was no time limit for the decision.

#### Questionnaires

German adaptations of the following questionnaires were applied: The Barratt Impulsiveness Scale [BIS-11; (24)] was used to obtain self-reported measures of impulsivity including subscales of motor impulsiveness, attention impulsiveness, and non-planning impulsiveness. In addition, we measured sensation seeking, which is correlated to risk taking and was also included in several studies investigating decision making and gambling (3, 16). Sensation seeking and the subscales thrill and adventure seeking, disinhibition, excitement seeking and boredom susceptibility were assessed with the Sensation Seeking Scale [SSS-V; (55)]. Additionally, participants completed the Alcohol Use Disorders Identification Test [AUDIT; (56)], the Beck Depression Inventory [BDI; (57)], the Fagerstrom Test for Nicotine Dependence [FTND; (58)], and the State-Trait Anxiety Inventory [STAI; (59)]. To quantify individual gambling behavior the South Oaks Gambling Screen [SOGS; (60)] was applied. A SOGS score of 5 or higher indicates probable PG.

#### Magnetic Resonance Imaging (MRI) Acquisition and Preprocessing

A high-resolution structural scan was acquired with a 3 Tesla Siemens Tim Trio MRI scanner and a 32-channel head coil using a sagittal T1-weighted MPRAGE sequence with following parameters: TR: 2,300 ms; TE: 2.91 ms; voxel size: 1 mm × 1 mm × 1.2 mm; slice thickness: 1.2 mm; field of view (FOV): 356 mm × 356 mm; 160 slices, flip angle: 9°.

Preprocessing of T1-weighted images was conducted with the Statistical Parametrical Mapping software (SPM12^1^) running in a Matlab 8.1 environment (Mathworks Inc.) using the Computational Anatomy Toolbox (CAT12^2^) and its default settings according to the CAT12 manual^3^. Preprocessing included normalizing using affine followed by non-linear registration, bias field correction, segmentation in gray matter, white matter, and cerebrospinal fluid (61) and normalization of the segmented scans into the Montreal Neurological Institute (MNI) space using Diffeomorphic Anatomic Registration Through Exponentiated Lie algebra algorithm [DARTEL; (62)]. The segmented, normalized images were smoothed with an 8-mm full width at half maximum (FWHM) Gaussian kernel.

In addition, we estimated cortical thickness with a fully automated method provided by CAT12 using an algorithm to calculate cortical surface parameters and projection-based thickness (63). Cortical thickness images were smoothed with a 15-mm FWHM Gaussian kernel.

### Procedure

Participants were invited to our research laboratory at the Christian-Doppler Medical Centre. After being informed about potential risks of MRI recordings participants provided informed written consent to take part in the experiment. For ethical reasons, neither the PG patients nor the controls received financial compensation for their participation in the study, but they were briefed that the goal of both tasks was to gain as much fictitious money as possible as if it was their own money. Since both tasks had no time limit for decision making, duration of task completion varied between participants (15–45 min.). The MRI session lasted approximately 60 min including the structural MRI recording and several fMRI tasks, which were not part of this study and will be reported in subsequent publications.

### Data Analysis

Degrees of freedom are not consistent over all analyses because some participants did not complete every task or questionnaire. To report effects sizes, we used Cohen's *d* for *t*-tests and *η* ^2^ for ANOVAs.

#### Decision Making

To analyze data of the cups task average percentage of risky choices was computed separately for every participant for the different EV levels and both domains. A mixed model ANOVA was conducted to analyze group differences and interactions between group and EV and/or domain. Further, EV sensitivity was calculated by subtracting percentage of risky choices in the risk-disadvantageous trials from the percentage of risky choices in risk-advantageous trials. Thus, it ranges from −1 (participant chooses every risk disadvantageous and no advantageous option) to +1 (participant chooses no risk disadvantageous and every advantageous option). EV sensitivity represents a parameter for advantageous decision making, because choosing the option with a favorable EV will yield more positive outcomes in the long run. Additionally, a risk-taking parameter was calculated by counting the overall number of risky choices. T-tests were used to analyze group differences in EV sensitivity and risk-taking.

Since education level and depression symptoms can have an influence on decision making abilities, we conducted additional analyses to control for group differences between PG patients and controls in these variables. We computed ANOVAs with EV sensitivity as dependent and group as independent variable and included years of education and the BDI score as covariates.

#### Impulsivity and Sensation Seeking

Data from the DDT was used to estimate an individual discounting parameter *K* using the R-package *hBayesDM* (64). The analysis was based on the standard hyperbolic model (65), where the subjective value of an reward option is determined by

(1) $$V=\frac{M}{\left(1+K*d\right)}$$

In this equation, *M* represents the magnitude of the monetary offer and *d* represents temporal delay. *K* determines the tendency to discount rewards further in the future in relation to sooner alternatives and reflects participant's behavioral impulsivity or the tendency to choose smaller-but-sooner (SBS) offers. We also counted the amount of SBS choices for every participant, because studies showed similar results for *K* and SBS choices (66). Two sample *t*-test were used to investigate group differences for behavioral impulsivity measures (*K* and SBS choices) as well as self-reported impulsivity (BIS) and sensation seeking (SSS) scores. To explore associations between deficient decision making and impulsivity or sensation seeking measures in the PG group, directional correlations were computed between EV sensitivity and *K*, SBS choices, and BIS and SSS scores.

#### Neuroimaging

Total intracranial volume (TIV) was estimated for every subject via CAT12 in order to use it as a covariate in VBM analyses. GMV differences between PG patients and controls were examined by submitting gray matter maps to a voxel-wise whole-brain two sample comparison in SPM12. To investigate the relationship between GMV and decision making in the cups task, a voxel-wise whole-brain regression model was computed in SPM12 including EV sensitivity, group, TIV and age. Further, the same analysis was computed separately for both groups using EV sensitivity, TIV, and age. Whole-brain analyses were conducted using family-wise error correction, *p* < 0.05, for multiple comparisons.

To particularly examine medial prefrontal regions, we selected several ROIs and estimated GMV in these ROIs by automated parcellation techniques as implemented in CAT12 using the Neuromorphometrics atlas (provided by Neuromorphometrics, Inc.^4^). First, we selected ROIs based on studies, which reported significant GMV differences between PG and control samples. Since both Zois et al. (40) and Ruiz de Lara et al. (41) reported reduced GMV in PG patients in relation to controls in the superior medial frontal gyrus (peaks at MNI coordinates: X = 0, Y = 57, Z = 12, and X = 6, Y = 42, Z = 42, respectively), we selected ROIs in the right and left superior medial frontal gyrus (sizes: 11,991 mm^3^ and 10,118 mm^3^, respectively). Secondly, based on our assumption, that the orbitofrontal cortex plays a essential role in calculating EVs in decision making (43), we also selected ROIs in the medial orbitofrontal cortex similar to the ventralmedial prefrontal ROIs from Bartra et al. (49). These ROIs included right and left medial orbital gyrus (sizes: 5,542 mm^3^ and 5,653 mm^3^, respectively), and right and left gyrus rectus (sizes: 2,926 mm^3^ and 3,078 mm^3^, respectively). See **Figure S1** for a depiction of all selected ROIs.

To comply with requests by reviewers, we conducted analyses examining white matter volume (WMV) and cortical thickness, and in addition, we investigated relations between neuroanatomical parameters and a performance parameter from the DDT, namely, the number of SBS choices. We did not investigate WMV in ROIs, because the Neuromorphometrics atlas does not provide ROIs for WMV. ROIs for cortical thickness were selected from a surface-based atlas to regionally correspond with the ROIs selected for GMV analyses. ROIs included right and left superior frontal gyrus [16]^5^, gyrus rectus [31], and medial orbital sulcus [63]. For more details on these ROIs, see Destrieux et al. (67).

Group differences in ROIs were evaluated using two-way ANOVAs. To analyze if there were different relations with decision making for the PG and control group, we first conducted regressions for every ROI with GMV as dependent variable, EV sensitivity as a main independent variable, and group as a moderation variable. Lastly, we computed correlations between EV sensitivity and GMV separately for PG patients and controls. In addition, we conducted the same ROI analysis procedure with SBS choices instead of EV sensitivity and also for cortical thickness measures. For all neuroimaging analyses, we used age as a covariate and for GMV and WMV analyses we also included TIV as covariate.

## Results

### Sample Characteristics

One participant from the control group was excluded from analysis because they had a score of 5 in the SOGS and therefore did not fulfill the requirements for the control group. Also, one participant from the PG group was excluded from analysis, because their average reaction time was shorter than 1 s in both tasks, which indicates that they were responding without assessing the different options before making their decision. Sample characteristics of the remaining participant is shown separated for PG patients and controls in **Table 2**. There was no significant difference between groups in sex ratio, age, tobacco (FTND), alcohol consumption (AUDIT), and overall brain volumes (TIV, GMV, WMV). Compared to the control group, the PG group had less average years of education, showed higher depression scores (BDI) and experienced higher state anxiety (STAI). As expected, there was a large difference in gambling behavior measured with the SOGS.

**Table 2:** Sample characteristics for control and PG group.

	Control group	PG group	Test statistics/Group differences
N/female	22/2	27/4	OR = 0.58, *p* = 0.678
Age	40.77 (14.29)	43.89 (11.89)	*t*(47) = −0.83, *p* = 0.409
TIV (ml)	1,555.8 (137.9)	1,555.1 (110.8)	*t*(45) = 0.02, *p* = 0.985; *d* = 0.00
GMV (ml)	698.1 (47.3)	693.5 (45.7)	*t*(45) = 0.33, *p* = 0.739; *d* = 0.10
WMV (ml)	547.8 (61.8)	556.4 (43.8)	*t*(45) = -0.55, *p* = 0.739; *d* = −0.16
Years of education	11.05 (1.56)	9.50 (1.39)	*t*(42.67) = 3.63, *p* = 0.001; *d* = 1.05*
FTND	0.95 (1.68)	1.58 (2.08)	*t*(46) = -1.13, *p* = 0.266; *d* = -0.33
AUDIT	3.71 (2.47)	4.45 (5.14)	*t*(41) = −0.60, *p* = 0.554; *d* = −0.18
BDI	5.09 (5.49)	13.92 (11.95)	*t*(36.35) = -3.37, *p* = 0.002; *d* = -0.92
STAI	35.65 (8.63)	45.24 (12.92)	*t*(40) = −2.68, *p* = 0.011; *d* = −0.84*
SOGS	0.14 (0.48)	9.88 (3.14)	*t*(26.43) = −15.59, *p* < 0.001; *d* = −4.12*

Values in parentheses represent standard deviation. FTDN, Fagerström Test for nicotine dependence; AUDIT, Alcohol Use Disorders Identification test; BDI, Beck Depression Inventory; STAI, State version of the State Rate Anxiety Inventory; SOGS, South Oaks Gambling Scale; *p < 0.05.

### Behavioral Results

#### Decision Making Group Differences

**Figure 2:**
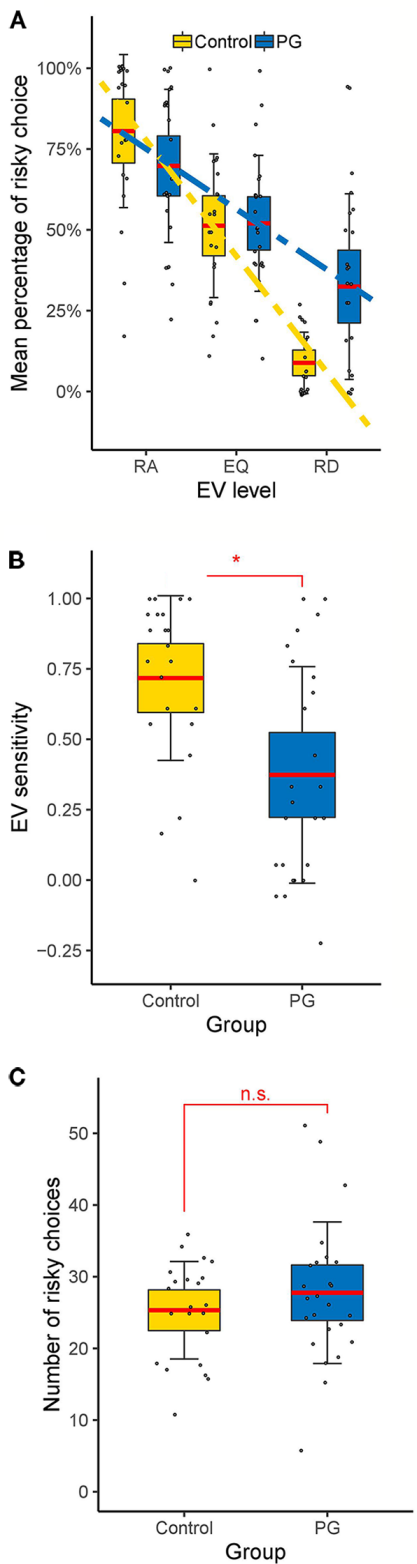
Behavioral results of the cups task. **(A)** Interaction between EV level and group. Differences between control and PG group in **(B)** EV sensitivity and **(C)** risky choices. Dotted lines show linear fits. Data points indicate individual participants. Thick red horizontal line mean; shaded regions ± error bars, 95% confidence intervals ± 1 s.d. of the mean for each condition and group. * *p* < 0.05 for two-sample *t*-test (two tailed). RA, risk advantageous; EQ, equal expected value; RD, risk disadvantageous; n.s., no significant difference (p > 0.05).

A 3 (EV level: risk advantageous/equal EV/risk disadvantageous) × 2 (Domain: gain/loss) × 2 (Group: PG/control) mixed model ANOVA with relative amount of risky choices as dependent variable was conducted. We found no group differences, *F*(1, 45) = 0.95, *p* = 0.335, but a large main effect for EV level, *F*(2, 67.16) = 89.75, *p* < 0.001, *η* ^2^ = 0.38. We also found a significant EV level × group interaction, *F* (2, 67.16) = 9.54, *p* = 0.001, *η* ^2^ = 0.06 (see **Figure 2A**). Post hoc between-group *t*-tests showed no significant difference in risk advantageous trials and trials with equal EV, *t*(45) < 1.57, *p* > 0.126, but a significant effect for risk disadvantageous trials, where PG patients choose the risky option more often than the control group, *t*(45) = −3.68, *p* = 0.001, *d* = −1.08.

Since we were not interested in differences between gain and loss trials, and there was no significant main effect for domain, *F*(1, 45) = 0.03, *p* = 0.856 nor any significant interactions between domain and other factors, *p* > 0.092, further analysis were conducted without separating for the domain factor. Thus, EV sensitivity and risk-taking were averaged over all gain and loss trials. PG patients showed significant less EV sensitivity than the control group (PG: *M* = 0.37; Controls: *M* = 0.72; See **Figure 2B**), *t*(45) = 3.41, *p* = 0.001, *d* = 1.00, but the groups did not differ in overall risk-taking (PG: *M* = 27.76; Controls: *M* = 25.32; See **Figure 2C**), *t*(45) = 0.97, *p* = 0.335, *d* = 0.28.

The difference in EV sensitivity between the PG and the control group remains significant when we included years of education (*F* (1, 45) = 4.85, *p* = 0.033, *η* ^2^ = 0.10) or BDI score (*F* (1, 44) = 6.02, *p* = 0.018, *η* ^2^ = 0.12) as covariates. There was no difference in response time between groups in the cups task (see **Figure S2**).

#### Impulsivity Group Differences

In the DDT, the larger-sooner reward was chosen dominantly (mean = 18.9 out of 20 catch trials), which indicates that the participants were concentrating and had their attention on the task. T-tests revealed (**Table 3**) that PG patients chose significantly more often the SBS option compared to the controls, and the difference in the discounting parameter *K* was numerically in the expected direction (in line with SBS) but did not reach statistical signifance (*p* = 0.090). There was no difference in response time between groups in the DDT (see **Figure S2**).

**Table 3:** Means (standard deviations) of impulsivity and sensation seeking measures and group differences between the control group and the PG group.

	Control group	PG group	Test statistics/Group differences
DDT			
K	0.10 (0.16)	0.18 (0.18)	*t*(44.99) = −1.73, *p* = 0.090, *d* = −0.50
SBS choices	69.00 (44.25)	96.68 (25.71)	*t*(32.80) = −2.58, *p* = 0.015, *d* = −0.78*
BIS- 11			
Attention	13.41 (2.00)	16.46 (3.92)	*t*(39.17) = −3.35, *p* = 0.002, *d* = −0.92*
Motor	21.12 (4.54)	22.96 (4.49)	*t*(41) = −1.31, *p* = 0.197, *d* = −0.41
Non-planning	20.71 (4.09)	27.15 (4.07)	*t*(41) = −5.07, *p* < 0.001, *d* = −1.58*
Total	55.24 (9.44)	66.50 (9.55)	*t*(41) = −3.80, *p* < 0.001, *d* = −1.18*
SSS			
TAS	6.47 (2.65)	4.46 (2.42)	*t*(41) = 2.56, *p* = 0.014, *d* = 0.80*
DIS	3.31 (2.09)	3.60 (2.55)	*t*(39) = −0.38, *p* = 0.708, *d* = −0.12
ES	6.53 (1.62)	4.35 (1.77)	*t*(41) = 4.01, *p* < 0.001, *d* = 1.28*
BS	3.29 (2.26)	3.23 (1.70)	*t*(41) = 0.10, *p* = 0.917, *d* = 0.03
Total	19.38 (6.00)	15.60 (5.53)	*t*(44) =2.22, *p* = 0.031, *d* = 0.66*

K, Discounting parameter; SBS choices, Smaller-but-sooner choices; TAS, Thrill and Adventure Seeking; DIS, Disinhibition; ES, Experience Seeking; BS, Boredom Susceptibility *p < 0.05.

Self-reported measures showed significant differences between the PG group and controls for every facet of impulsivity except motor impulsivity (**Table 3**). And as reported in **Table 3**, there were also significant differences in sensation seeking subscales “Thrill and adventure seeking” and “Experience seeking” as well as in the total sensation seeking score.

#### Correlation Between Decision Making and Impulsivity

Correlations in PG participants between EV sensitivity and several facets of impulsivity and sensation seeking measures are depicted in **Table 4**. Out of the cognitive impulsivity measures, the non-planning impulsivity score from the BIS-11 was negatively correlated with EV sensitivity (*r* = −0.37, *p* = 0.039), while both measures for delay discounting showed no significant association to EV sensitivity. No other facet of impulsivity or sensation seeking revealed a significant relationship to EV sensitivity in the PG group, but the attentional impulsivity facet showed a relatively strong but not significant relationship (*r* = −0.32, *p* = 0.064).

**Table 4:** Correlation coefficients and corresponding P-values (one-sided) for relations between EV sensitivity and several impulsivity and sensation seeking measures.

	EV sensitivity	
	*r*	*p*
Delay discounting task		
*K*	0.28	0.904
SBS choices	0.10	0.669
Barrant Impulsiveness Scale		
Attention subscale	−0.32	0.064
Motor subscale	−0.04	0.426
Non-planning subscale	−0.37	0.039 *
Sensation Seeking Scale		
Thrill and adventure subscale	0.16	0.789
Disinhibition subscale	−0.03	0.452
Experience subscale	−0.04	0.422
Boredom susceptibility sub.	−0.21	0.165

K, Discounting parameter; SBS, Smaller-but-sooner; *p < 0.05 (one sided).

### Neuroimaging Results

#### Group Differences

Data from 25 PG patients and 22 controls was compared for these analyses. No GMV difference between PG and control group was found in the whole-brain analysis. Two-way ANOVAs with TIV and age as covariates showed also no significant GMV difference between PG and control group in the prefrontal ROIs (see **Table 5**). Whole-brain analyses for WMV and cortical thickness also showed no group differences, and there were no significant differences in cortical thickness measures between PG patients and controls in the surface-based ROIs (see **Table S1**).

**Table 5:** Group differences for GMV between PG patients (n = 25) and controls (n = 22) in predefined prefrontal ROIs.

ROI	*F*	*p*	*η^2^*
L medial superior frontal gyrus	0.85	0.361	0.02
R medial superior frontal gyrus	0.96	0.333	0.02
L medial orbital gyrus	1.96	0.169	0.04
R medial orbital gyrus	0.95	0.336	0.02
L gyrus rectus	2.39	0.129	0.05
R gyrus rectus	0.00	0.978	0.00

L, left; R, right; Covariates included TIV and age.

#### Relations Between Decision Making and GMV

To find relations between decision making performance and GMV, data from 24 PG patients and 22 controls was available, but two patients did not complete the cups task and two other patients did not complete the DDT, which leads to 22 PG patients for each task. First, the whole brain analysis revealed no significant interactions between EV sensitivity and group regarding GMV, WMV, or cortical thickness. Secondly, there were also no significant interactions between number of SBS choices in the DDT and group regarding GMV, WMV, or cortical thickness on a whole brain level.

Although there were no interactions between EV sensitivity and group in GMV ROIs (see **Table 6**), we found positive correlations between EV sensitivity and GMV in the right and left gyrus rectus in PG patients (*r* = 0.58, *p* = 0.007, and *r* = 0.52, *p* = 0.020, respectively), indicating that increased GMV in these regions corresponds with higher EV sensitivity and thus better decision making. No further significant correlations were revealed neither in patients nor in controls (**Table 7**).

**Table 6:** Regression results for GMV in predefined prefrontal ROIs.

ROI	Coeffecients	β	*p*
L medial superior frontal gyrus	EV sensitivity	0.15	0.512
	EV sensitivity × Group	−0.22	0.423
R medial superior frontal gyrus	EV sensitivity	0.07	0.795
	EV sensitivity × Group	−0.04	0.901
L medial orbital gyrus	EV sensitivity	−0.41	0.115
	EV sensitivity × Group	0.54	0.087
R medial orbital gyrus	EV sensitivity	−0.27	0.313
	EV sensitivity × Group	0.51	0.113
L gyrus rectus	EV sensitivity	−0.10	0.651
	EV sensitivity × Group	0.46	0.093
R gyrus rectus	EV sensitivity	0.12	0.602
	EV sensitivity × Group	0.39	0.182

L, left; R, right; Covariates included TIV and age; Controls: n = 22; PG: n = 22.

**Table 7:** Correlation coefficients and corresponding P-values for relations between EV sensitivity and GMV in predefined ROIs.

Group	ROI	EV sensitivity	
		*r*	*p*
Controls	L medial superior frontal gyrus	0.08	0.732
n = 22	R medial superior frontal gyrus	0.06	0.815
	L medial orbital gyrus	−0.36	0.115
	R medial orbital gyrus	−0.24	0.312
	L gyrus rectus	−0.33	0.152
	R gyrus rectus	−0.12	0.614
PG	L medial superior frontal gyrus	−0.14	0.545
n = 22	R medial superior frontal gyrus	0.04	0.867
	L medial orbital gyrus	0.17	0.483
	R medial orbital gyrus	0.33	0.150
	L gyrus rectus	0.52	0.020 *
	R gyrus rectus	0.58	0.007 *

L, left; R, Right; *p < 0.05.

Examining the relations between DDT and GMV we found no interaction for number of SBS choices and group (see **Table S2**). Also, there were no significant correlations between SBS choices and GMV for PG patients, but a negative relationship was found in controls in the left medial orbital gyrus, *r* = −0.45, *p* = 0.049 (see **Table S4**), which means that increased GMV in this area relates to lower number of SBS choices and thus lower impulsivity.

Regression analyses with EV sensitivity in cortical thickness ROIs revealed a negative interaction between EV sensitivity and group in the right medial orbital sulcus, *β* = −.82, *p* = 0.024 (see **Table S3**). However, there were no significant correlations neither in the PG nor the control group (see **Table S5**). Furthermore, we found no interaction between SBS choices and group for cortical thickness, but a significant main effect for SBS choices in the left gyrus rectus, *β* = −.52, *p* = 0.004 (see **Table S3**). This main effect was also found in results for correlations between SBS choices and cortical thickness in the left gyrus rectus both in PG patients (*r* = −0.48, *p* = 0.029) and in controls (*r* = −0.52, *p* = 0.016; See **Tables S5**).

## Discussion

In this study, we investigated deficient decision making in PG patients and its connection to impulsive personality traits and neuroanatomical characteristics. Two questions were raised: Does poorer decision making correlate with higher impulsivity in patients suffering from PG? And is there a relation between GMV and decision making measures in PG patients?

### Association Between Decision Making and Impulsivity in PG

As predicted, based on the majority of findings on impulsivity and decision making in PG (3, 5, 6, 10–12), our results showed that PG patients exhibit impaired decision making and multiple increased impulsivity dimensions compared to a control group. In line with non-significant differences in self-reported motor impulsivity, we also did not find differences in response time in both the cups task and the DDT. Regarding an association between decision making and impulsivity, we assumed in line with Kräplin et al. (17), that cognitive rather than motor of impulsivity would be correlated with decision making. Accordingly, results from the PG sample revealed a negative correlation between the subscale of non-planning impulsivity (but not motor impulsivity) and the risk evaluation component of decision making, thus conforming our first main hypothesis.

Contrary to Kräplin et al. (17), the current study found no association between decision making and delay discounting, which could be explained by the usage of different decision making measures. Kräplin et al. found a correlation between delay discounting and a risk seeking component of decision making, whereas we investigated risk evaluation instead, which may have different relations to delay discounting. Additionally, although both the non-planning subscale and delay discounting are regarded as cognitive impulsivity measures, self-reporting, and behavioral approaches to measure impulsivity can lead to different results (25).

In sum, these findings illustrate the benefit of a multi-dimensional approach when investigating impulsivity. Since different impulsivity dimensions may arise from different processes, not all show similar connections to other traits (68). Our results suggest, that higher cognitive impulsivity and impaired decision making in PG patients result both from deficient valuation-related processes. An explanation might be, that PG causes altered valuation-related processes (69), leading to deficient decision making and negative outcomes, and in turn leading to PG patients becoming more impulsive in regard to planning ahead because they learned that deliberate decision making is inefficient. However, a longitudinal study design would be necessary, to draw clear conclusions on potential causal relations.

### Association Between Decision Making and GMV in PG

First of all, in contrast to recent findings (37, 40, 41), we did not find any brain regions with differences in GMV between the PG and the control group. Thus, our hypothesis about GMV differences could not be confirmed. This result is also in accordance with the study by van Holst et al. (70), who compared GMV from PG patients, patients suffering from alcohol use disorder and a HC group. They argued, that the effect of PG on GMV seems to point in the same direction as the effect of alcohol use disorder but it is smaller and therefore needs a larger sample size to yield significant volume differences. Since we have a smaller sample size than other recent studies reporting GMV differences, this limitation may also explain our findings.

Regarding our second assumption concerning the relation of GMV and decision making, we did not find a significant interaction between PG and control group, but our results revealed a positive correlation between GMV and EV sensitivity in PG patients in bilateral areas of the medial orbitofrontal cortex. Thus, confirming our second main hypothesis, reduced volume of gray matter in the orbitofrontal cortex is related to poorer decision making in patients suffering from PG. Analogous to Tanabe et al. (35), who reported a correlation between orbitofrontal gray matter and decision making in a sample of abstinent substance-dependent individuals, our findings support the assumption, that alterations in the orbitofrontal cortex play a crucial role in deficient reward processing.

One study also reported decreased GMV in the orbitofrontal cortex in participants with higher non-planing impulsivity (47). Since our results regarding decision making and impulsivity indicate that increased non-planing impulsivity is correlated with reduced EV sensitivity, these findings by Matsuo et al. (47) would also indirectly support our assumption that orbitofrontal GMV is connected to outcome evaluation abilities.

Additional analyses regarding GMV and cortical thickness measures in relation to delay discouting also support the important role of the medial orbitofrontal cortex in the context of poor decision making. Reduced GMV and cortical thickness in the medial orbitofrontal cortex are not just connected to low EV sensitivity but also increased delay discounting (71).

### Additional Remarks on Our Findings on Decision Making in PG

The majority of studies using the cups task reported differences in decision making between the gain and loss domain (44, 72, 73), which is in line with the concept of loss aversion, that explains that people are more sensitive to the possibility of losing money than to the possibility of winning the same amount of money (74). In contrast, we found no decision making difference between the gain and loss domain neither in the gambling nor in the control group. Although there are studies reporting reduced loss aversion in PG (75), our results can probably best be explained by two specific changes in our adaptation of the cups task, which made participants care less if outcomes are gains or losses. One change was, that we only used hypothetical rewards. But some studies, which reported differences in the gain/loss domain, also used hypothetical rewards (72). However, we also did not display any outcomes at the end of each trial. So, even if participants would try to maximize their hypothetical gain (or minimize their hypothetical loss), they would not know how much they actually gained (or lost) each trial, and thus, the task would seem less like one big game and more like independent choices that have nothing to do with each other. In sum, these methodical alterations should explain the non-existing gain/loss domain effect.

Furthermore, we found no difference in the amount of risky choices between gamblers and controls. This result is contrary to most studies on PG, which report increased risky taking in PG patients (6, 17, 76). One could argue, that the methodical changes in our task design also play a role in the missing risk taking effect, because it reduces the motivation to engage in risky behavior. However, we still found a relatively strong effect in risk evaluation, indicating that PG patients' deficits in decision making are primarily connected to risk evaluation, not risk seeking, which also is in line with our neuroanatomical findings regarding reduced GMV in the orbitofrontal cortex (43).

### Limitations

First, in the current study, we did not distinguish between varieties of PG. Studies have shown that casino gamblers, who mostly play card games, act more strategic and exhibit less decision making deficits compared to slot machine gamblers (77). Our sample was too small to reasonably split it into subgroups of PG, but we would encourage future studies to investigate the relations of decision making and neuroanatomical characteristics separately for different gambling types, because we would expect clearer results, e.g., regarding GMV differences between HCs and non-strategic gamblers. Secondly, although we had reasonable sample sizes, the samples were still too small for correlations between GMV and EV with moderate effect sizes to yield significant results. Thirdly, the PG and control group differed in their years of education and depression scores. On the one hand, some studies found no significant influence of education on impaired decision making (29). On the other hand, there are also studies showing that education-related skills like numeracy have an effect on decision making in the cups task (78). Therefore, we also showed that the decision making differences between both groups remained, when controlling for education and BDI scores.

## Conclusion

Our study investigated the relations of decision making with impulsivity traits as well as GMV in pathological gamblers. Results showed an association between decision making deficits and specific impulsivity facets in PG patients, which suggests that necessity to consider multiple dimensions, when investigating impulsivity in a PG sample. Secondly, our findings revealed that dysfunctional decision making—particularly, the component of risk evaluation—is related to reduced GMV in the medial orbitofrontal cortex, a brain region connected with processing EVs of potential outcomes. Interestingly, PG patients and controls did not differ in risky choices, and thus, we assume that decision making deficits in PG are primarily related to risk evaluation, not risk seeking, which is in line with our GMV findings.

## Data Availability Statement

The datasets generated for this study are available on request to the corresponding author.

## Ethics Statement

The studies involving human participants were reviewed and approved by the ethic commitee of the Federal State Salzburg. The patients/participants provided their written informed consent to participate in this study.

## Author Contributions

DF drafted the original manuscript and carried out behavioral data analyses. DF and MK performed voxel-based morphometry analyses. FW, NT, and WA were responsible for diagnosing and recruiting participants. ML, PS, TE, and MK were responsible for data collection. DF and MK participated in result interpretation and discussion, and in successive revisions of the original manuscript. MK and FW are the principal investigators of the research project.

## Funding

This work was funded by grants provided to MK by the Austrian Science Fund (FWF grant number: P30390-B27). DF was in addition financially supported by the Doctoral College “Imaging the Mind” at the University of Salzburg (FWF; W1233).

## Conflict of Interest

The authors declare that the research was conducted in the absence of any commercial or financial relationships that could be construed as a potential conflict of interest.

## Footnotes

1
http://www.fil.ion.ucl.ac.uk/spm/software/spm12/

2
http://dbm.neuro.uni-jena.de/cat/

3
http://www.neuro.uni-jena.de/cat12/CAT12-Manual.pdf

4
http://Neuromorphometrics.com

5 Numbers in squared brackets refer to the index in the surface-based atlas as provided by Destrieux et al. (67)
