# Supplementary material for: Deficient Decision Making in Pathological Gamblers Correlates With Gray Matter Volume in Medial Orbitofrontal Cortex
Source: Front Psychiatry. 2020 Mar 4;11:109. doi: 10.3389/fpsyt.2020.00109 (PMC7064713; doi:10.3389/fpsyt.2020.00109)
Supplement: Supplementary file 1 [file DataSheet_1.pdf]

Supplementary Material

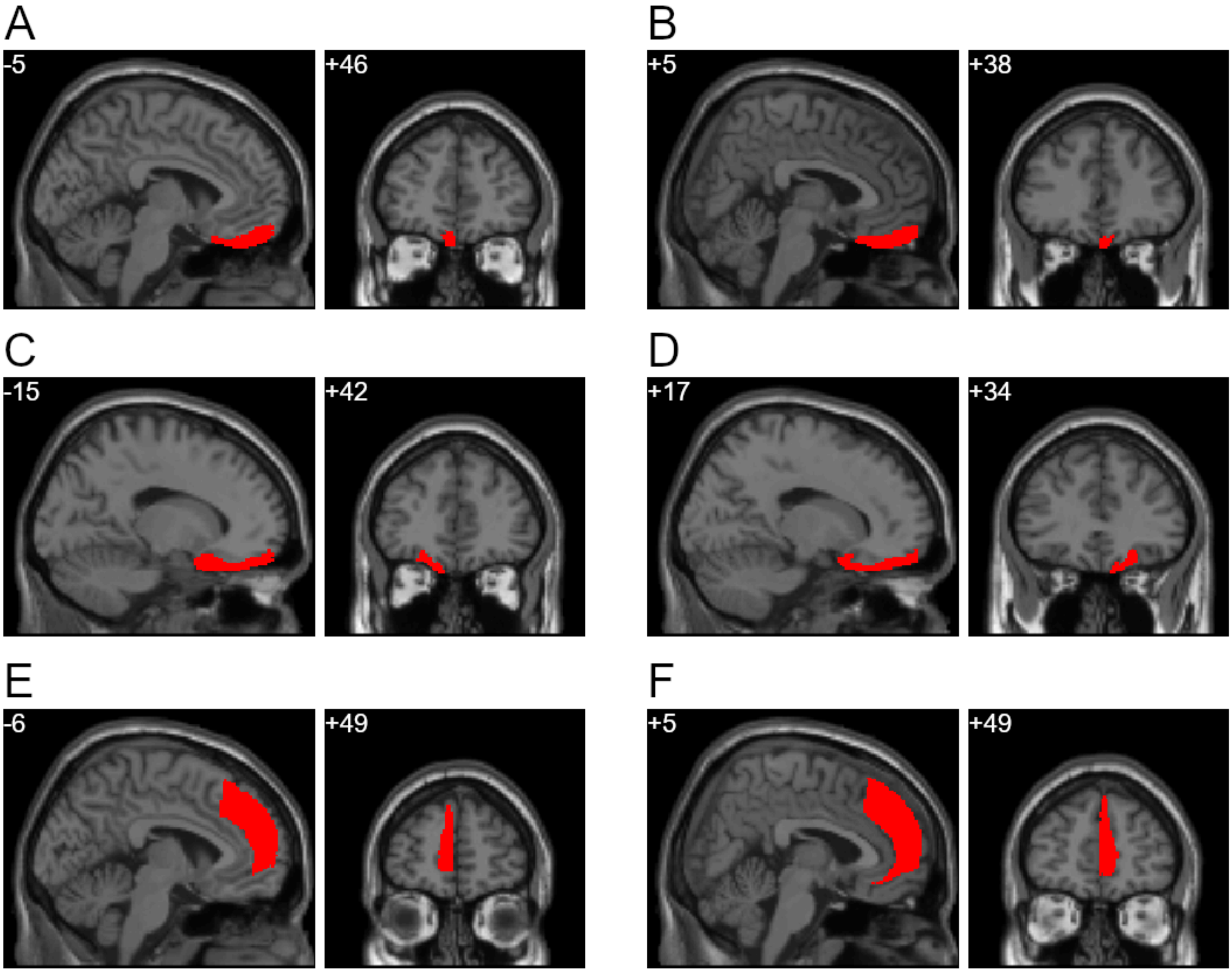

Figure S1. Regions of interest (ROIs) for gray matter volume (GMV) analyses included (A) left rectus gyrus (3078 mm^3), (B) right rectus gyrus (2926 mm^3), (C) left medial orbital gyrus (5653 mm^3), (D) right medial orbital gyrus (5542 mm^3), (E) left medial superior frontal gyrus (10118 mm^3) and (F) right medial superior frontal gyrus (11991 mm^3) as defined in the Neuromorphometrics atlas (provided by Neuromorphometrics, Inc.).

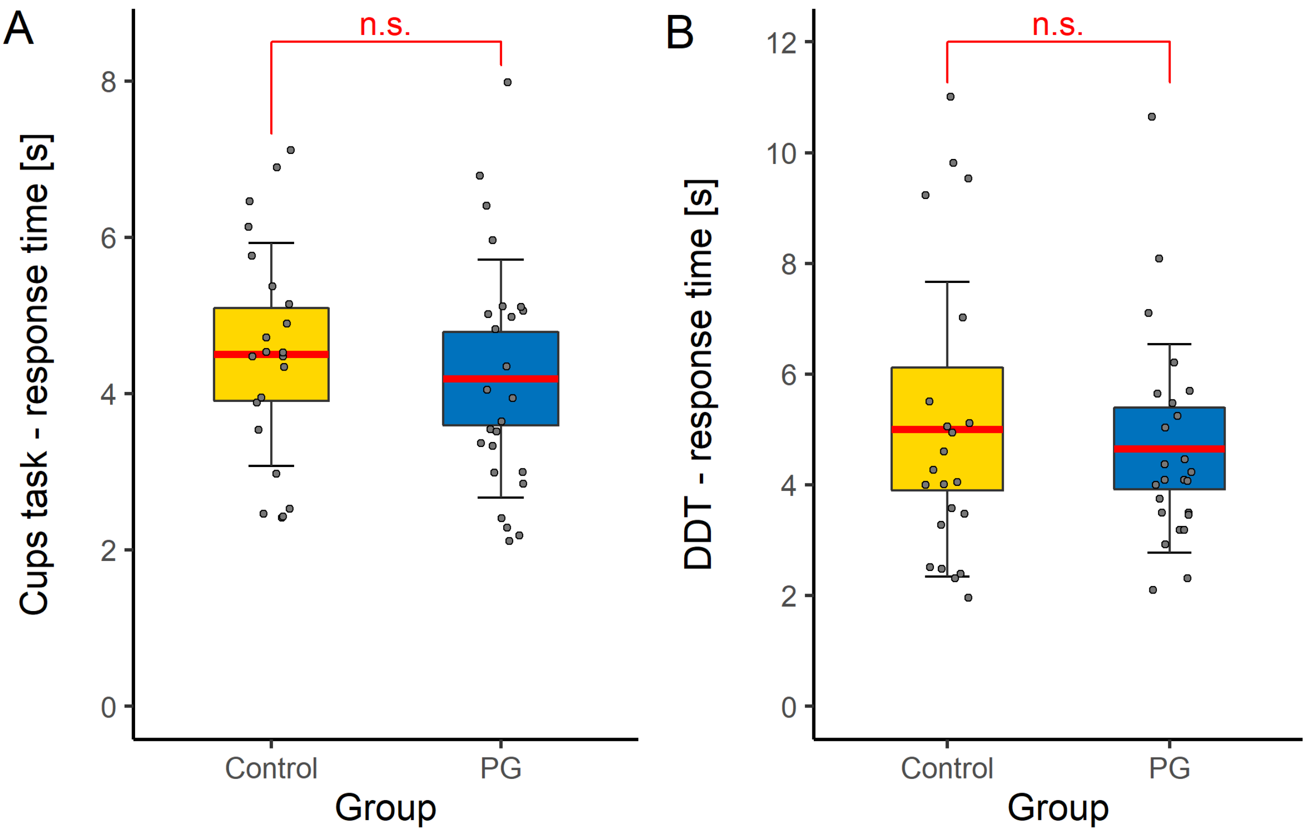

Figure S2. Response times did not differ between groups in the (A) cups task (Controls: $M = 4.50$ s; PG: $M = 4.19$ s; $t(45) = 0.71$, $p = 0.478$, $d = 0.21$) and (B) the DDT (Controls: $M = 5.01$ s; PG: $M = 4.66$ s; $t(45) = 0.53$, $p = 0.602$, $d = 0.15$).

Table S1. Group differences for cortical thickness between PG patients (n = 25) and controls (n = 22) in predefined prefrontal ROIs.

ROI	*F*	*p*	η^2
L superior frontal gyrus	1.55	0.220	0.03
R superior frontal gyrus	1.41	0.242	0.03
L medial orbital sulcus	2.25	0.141	0.05
R medial orbital sulcus	0.25	0.616	0.01
L gyrus rectus	0.00	0.984	0.00
R gyrus rectus	0.08	0.784	0.00

Notes. L = left; R = right; Covariates included age.

Table S2. Regression results for GMV in predefined prefrontal ROIs.

ROI	Coefficients	β	*p*
L medial superior frontal gyrus	SBS	-0.04	0.774
	SBS x Group	0.07	0.818
R medial superior frontal gyrus	SBS	-0.17	0.291
	SBS x Group	0.12	0.708
L medial orbital gyrus	SBS	0.02	0.905
	SBS x Group	-0.54	0.114
R medial orbital gyrus	SBS	-0.13	0.424
	SBS x Group	-0.22	0.522
L gyrus rectus	SBS	0.14	0.371
	SBS x Group	-0.13	0.676
R gyrus rectus	SBS	-0.09	0.592
	SBS x Group	0.17	0.622

Notes. L = left; R = right; Covariates included TIV and age; Controls: n = 22; PG: n = 22.

Table S3. Regression results for cortical thickness in predefined prefrontal ROIs.

	ROI	Coefficients	β	*p*
EV sensitivity	L superior frontal gyrus	EV sensitivity	-0.34	0.179
		EV sensitivity x Group	0.53	0.091
	R superior frontal gyrus	EV sensitivity	-0.31	0.218
		EV sensitivity x Group	0.56	0.073
	L medial orbital sulcus	EV sensitivity	-0.02	0.946
		EV sensitivity x Group	0.02	0.950
	R medial orbital sulcus	EV sensitivity	0.41	0.162
		EV sensitivity x Group	-0.82	0.024 *
	L gyrus rectus	EV sensitivity	-0.30	0.322
		EV sensitivity x Group	0.01	0.975
	R gyrus rectus	EV sensitivity	-0.14	0.628
		EV sensitivity x Group	0.00	0.993
SBS	L superior frontal gyrus	SBS	0.02	0.929
		SBS x Group	0.03	0.939
	R superior frontal gyrus	SBS	0.14	0.406
		SBS x Group	0.05	0.887
	L medial orbital sulcus	SBS	-0.13	0.465
		SBS x Group	0.31	0.404
	R medial orbital sulcus	SBS	-0.35	0.069
		SBS x Group	0.19	0.620
	L gyrus rectus	SBS	-0.52	0.004 *
		SBS x Group	-0.07	0.850
	R gyrus rectus	SBS	-0.22	0.235
		SBS x Group	-0.24	0.522

Notes. L = left; R = right; Covariates included age; Controls n = 22; PG n = 22; * $p < 0.05$

Table S4. Correlations between GMV in predefined prefrontal ROIs and SBS choices separately for PG patients and controls.

		SBS choices	
Group	ROI	*r*	*p*
Controls	L medial superior frontal gyrus	-0.03	0.916
n = 22	R medial superior frontal gyrus	-0.26	0.273
	L medial orbital gyrus	0.02	0.939
	R medial orbital gyrus	-0.17	0.475
	L gyrus rectus	0.29	0.209
	R gyrus rectus	-0.08	0.731
PG	L medial superior frontal gyrus	0.01	0.973
n = 22	R medial superior frontal gyrus	-0.01	0.971
	L medial orbital gyrus	-0.45	0.049 *
	R medial orbital gyrus	-0.31	0.177
	L gyrus rectus	0.02	0.930
	R gyrus rectus	0.05	0.818

Notes. L = left; R = right; Covariates included TIV and age; * $p < 0.05$

Table S5. Correlations between cortical thickness in predefined prefrontal ROIs and EV sensitivity or SBS choices separately for PG patients and controls.

		EV sensitivity	
Group	ROI	*r*	*p*
Controls	L superior frontal gyrus	-0.29	0.203
n = 22	R superior frontal gyrus	-0.26	0.251
	L medial orbital sulcus	0.05	0.844
	R medial orbital sulcus	0.31	0.170
	L gyrus rectus	-0.21	0.367
	R gyrus rectus	-0.11	0.650
PG	L superior frontal gyrus	0.31	0.169
n = 22	R superior frontal gyrus	0.37	0.097
	L medial orbital sulcus	-0.01	0.950
	R medial orbital sulcus	-0.34	0.129
	L gyrus rectus	-0.32	0.153
	R gyrus rectus	-0.14	0.557

		SBS choices	
Group	ROI	*r*	*p*
Controls	L superior frontal gyrus	0.02	0.918
n = 22	R superior frontal gyrus	0.17	0.466
	L medial orbital sulcus	-0.21	0.361
	R medial orbital sulcus	-0.40	0.074
	L gyrus rectus	-0.52	0.016 *
	R gyrus rectus	-0.31	0.169
PG	L superior frontal gyrus	0.06	0.808
n = 22	R superior frontal gyrus	0.17	0.453
	L medial orbital sulcus	0.08	0.726
	R medial orbital sulcus	-0.04	0.852
	L gyrus rectus	-0.48	0.029 *
	R gyrus rectus	-0.28	0.217

Notes. L = left; R = right; Covariates included age; * $p < 0.05$
